# Hypertrophic Pachymeningitis in a Southern Chinese Population: A Retrospective Study

**DOI:** 10.3389/fneur.2020.565088

**Published:** 2020-11-17

**Authors:** Xuewen Xiao, Dongni Fu, Li Feng

**Affiliations:** ^1^Department of Neurology, Xiangya Hospital, Central South University, Changsha, China; ^2^Department of Gastroenterology, Xiangya Hospital, Central South University, Changsha, China

**Keywords:** hypertrophic pachymeningitis (HP), retrospective study, Chinese, clinical features, neuroimaging

## Abstract

**Aims:** To investigate the causes, clinical characteristics, imaging features, and therapeutic implications of hypertrophic pachymeningitis (HP) in a southern Chinese population.

**Methods:** We retrospectively analyzed 48 patients with HP with different causes from 1 January 2006 to 31 December 2018. Clinical manifestation, laboratory findings, and neuroimaging results were evaluated in all HP patients.

**Results:** The mean age at onset was 50 ± 12 years. The most common diagnosis was idiopathic HP (67%), followed by antineutrophil cytoplasmic antibody (ANCA)-associated vasculitis (15%), tuberculous meningitis (8%), viral meningitis (6%), and bacterial meningitis (4%). Headache was the most common symptom. The most frequently changed laboratory finding was elevated erythrocyte sedimentation rate (ESR). Imaging was characterized by cerebral or spinal dura mater enhancement in MRI scan with contrast. Enhancements were mainly located in the posterior fossa for idiopathic HP; frontal, parietal, and occipital lobes for ANCA-related HP; and posterior fossa for tuberculous-associated HP. Diffuse enhancement was found in most cases, except for tuberculous-associated HP. Glucocorticoid or immunosuppressive treatment was applied in most cases.

**Conclusions:** The etiology of HP varied among patients, with idiopathic HP being the most common. MRI showed enhancement of the dura mater, which differed according to different etiologies. Glucocorticoid or immunosuppressive agents were the primary drugs for treatment.

## Introduction

Hypertrophic pachymeningitis (HP) is a fibrosing inflammatory disorder featuring localized or diffused thickening of the cranial or spinal dura mater. Enhanced MRI is currently the most powerful tool to diagnose HP. The disorder can be divided into cranial or spinal pachymeningitis by lesion location and idiopathic or secondary HP by etiology. Infections and autoimmune diseases are among the most identified causes of secondary HP (1). Notably, the thickening of the dura mater is present in other conditions, such as intracranial hypotension syndrome or neoplastic pachymeningitis, which should be carefully differentiated to avoid misdiagnosis.

As a rare disease, the prevalence of HP has been reported to be 0.949/100,000 persons, half of whom had idiopathic HP (2). Such a low prevalence contributes to the fact that most published articles are case reports, which has made it difficult to draw a complete picture of the disease. Furthermore, most HP cases have been reported in the Caucasian population; only a few studies of HP, with relatively small sample sizes, are available in China (3–5). In this study, we retrospectively investigated 48 patients diagnosed with HP in our neurology department. With the largest sample size in HP patients thus far, we hope to characterize HP in terms of its etiology and clinical manifestation in a southern Chinese population and further our understanding of HP.

## Methods

Forty-eight patients diagnosed with HP were retrospectively reviewed from 1 January 2006 to 31 December 2018 in the neurology department of Xiangya Hospital from electronic medical records. The protocol of the present study was approved by the ethics committee of Xiangya Hospital, Central South University, and written informed consent was obtained from the adult participants or their parents. The entry criteria were defined as follows: (i) diagnosis was based on dura mater biopsy or dura mater enhancement in gadolinium MRI T1 sequences, and (ii) dural thickening could not be explained by intracranial hypotension, neoplastic pachymeningitis, or other conditions. We collected clinical, laboratory, imaging, and therapeutic data from these patients from our electronic medical records system.

MRI enhancements were categorized by their locations, distributions, and patterns according to a definition described by Hahn et al. (6). Locations were identified by an experienced radiologist. Distributions were categorized according to the following standards: lesions covering more than 50% of the intracranial compartment or greater than five vertebral levels were defined as “diffuse enhancement,” and the rest were described as “focal enhancement.” Furthermore, we used the concept of “roughness” to describe the enhancement patterns in a more detailed manner. “Roughness” was classified into “irregular” and “regular.” “Irregular” referred to the existence of nodules, whereas “regular” was characterized by linear enhancement without any nodules.

Clinical records, including basic information, such as age at onset, sex, and duration; clinical presentations, which were recorded from the history of the present illness; and laboratory findings, such as white blood cell (WBC) count, neutrophil count, erythrocyte sedimentation rate (ESR), C-reactive protein (CRP), rheumatoid factor (RF), cerebrospinal fluid (CSF) pressure, CSF protein, CSF glucose, CSF adenosine deaminase (ADA), CSF immune globulin, and other laboratory results during the hospitalized period, were collected.

## Results

### Demographics

**Figure 1:**
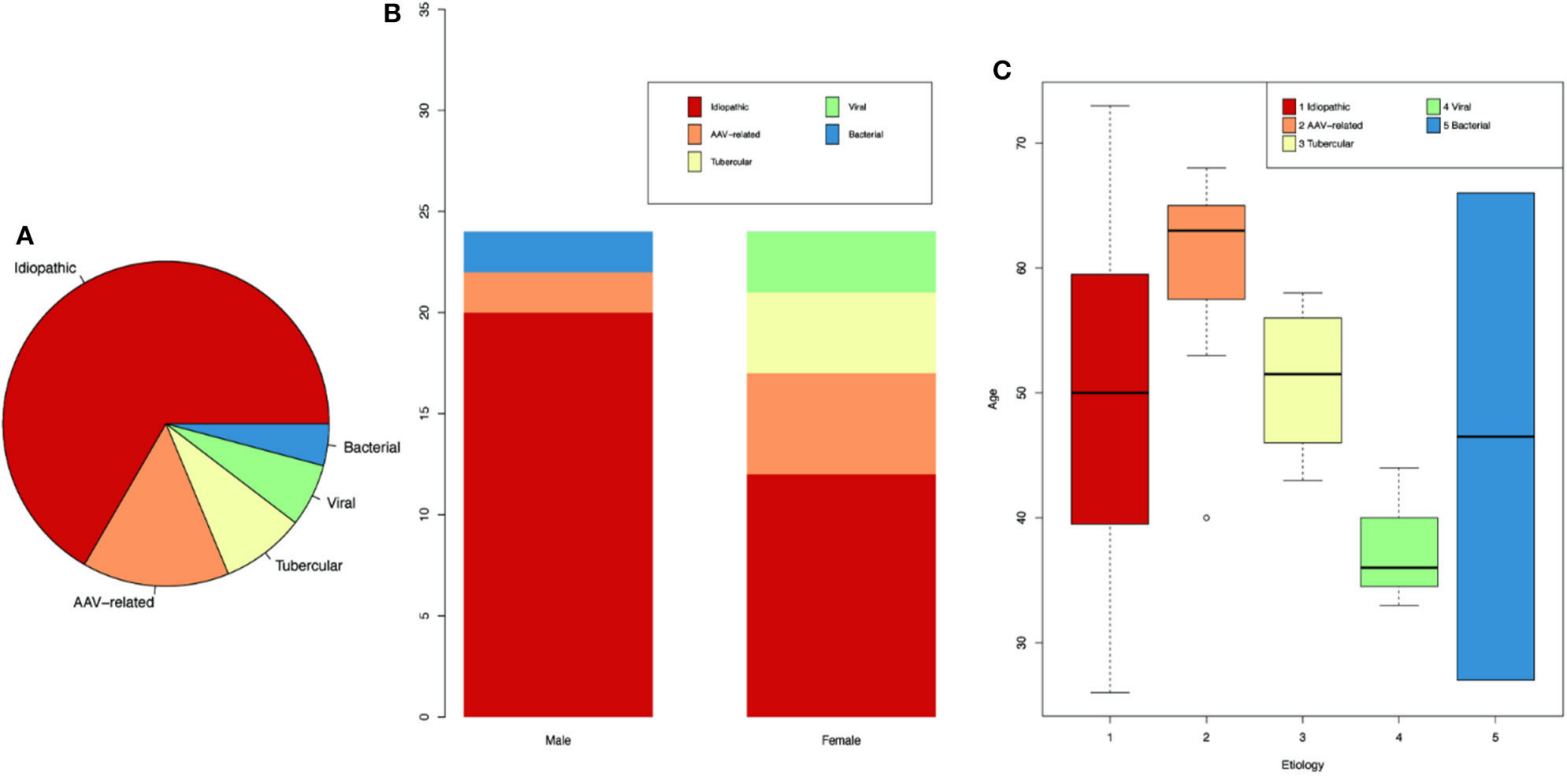
Demographics for HP patients of different etiologies. **(A)** Proportion of HP patients according to different etiologies. **(B)** Number of patients according to genders. **(C)** Age of onset for patients of different etiologies.

Among the 48 HP cases, there were 24 men and 24 women, with no sex predominance. However, the distribution of etiology differs between genders. Idiopathic and antineutrophil cytoplasmic antibody (ANCA)-associated vasculitis (AAV)-related HP were found in both genders, whereas viral and tubercular HP were only found in female, and bacterial HP was only found in males. The most common diagnosis was idiopathic HP (32/48, 67%), followed by AAV (7/48, 15%), tuberculous meningitis (4/48, 8%), viral meningitis (3/48, 6%), and bacterial meningitis (BM) (2/48, 4%) (Supplementary Table 1 and Figures 1A,B). The first HP-related symptom occurred at an average age of 50 ± 12 years (range 26–73 years). Among the HP patients, those with AAV had the oldest age at onset (60 ± 13 years), and the youngest were those with viral meningitis, whose average age at onset was 38 ± 12 years (Figure 1C).

The duration from symptom onset to diagnosis of HP ranged from 0.25 to 144 months (with an average of 22 months), among which BM had the shortest duration (0.6 ± 0.4 months), whereas idiopathic HP had the longest duration (25 ± 36 months).

### Radiographic Imaging Features

**Figure 2:**
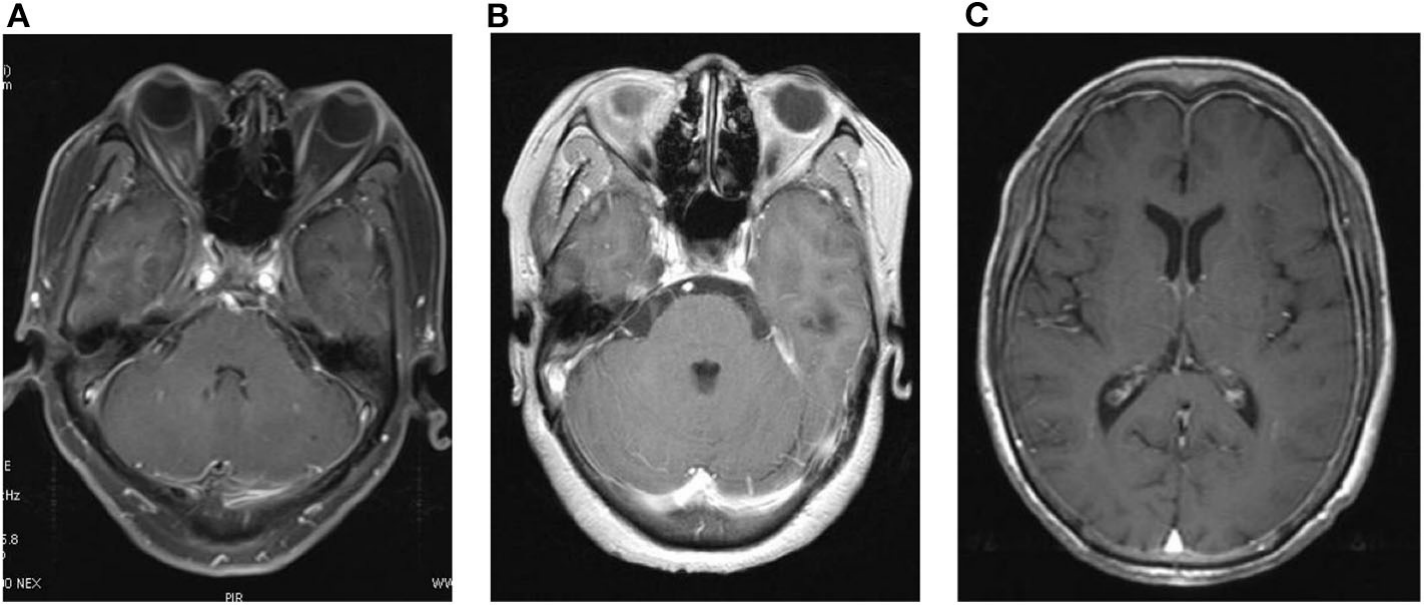
Typical radiology features for HP patients. **(A)** A 61 year-old man presented with headache and hearing loss. He was diagnosed with idiopathic HP. There was dural enhancement of the posterior fossa in an axial T1-weighted MRI. **(B)** Axial T1-weighted MRI of a 54 year-old woman demonstrated dural enhancement of the posterior fossa. She was diagnosed with tuberculous-related HP. **(C)** A 66 year-old woman complained of headache, right hearing loss, and right vision loss for 2 months. MPO antibodies were positive, and a diagnosis of ANCA-related HP was established. The enhanced MRI showed dural enhancement of the frontal, parietal, and occipital lobes.

Locations of the enhancements varied greatly among the different types of HP. The posterior fossa was the most affected area in idiopathic HP (15 cases; Figure 2A) and was also common in patients with tuberculous meningitis HP (3 cases; Figure 2B), whereas the frontal, parietal, and occipital lobes were the most affected areas in AAV (3 cases; Figure 2C).

For the enhancement distribution patterns, we found that idiopathic (16/32), AAV (4/7), and viral HP (2/3) presented diffuse enhancement patterns. In contrast, almost all tuberculous meningitis HP (4/4) showed focal enhancement patterns. “Irregular” enhancement was much more common in idiopathic HP patients (15/32), whereas other types of HP exhibited more regular patterns (Supplementary Table 2).

### Clinical Features

Headache, the most common clinical feature of HP, regardless of etiology, occurred in 92% of all HP patients (Supplementary Table 1) and accounted for 94% of idiopathic HP (30/32), 100% of AAV HP (7/7), 75% of tuberculous meningitis HP (3/4), 100% of viral meningitis HP (3/3), and 50% of BM HP (1/2).

Cranial nerve deficits, the second most common symptom, existed in more than half of the idiopathic HP, AAV, and tuberculous meningitis patients. In cases of idiopathic HP, cranial nerve II (optic nerve) was the most frequently affected nerve (25%), followed by cranial nerves VI, VIII, III, IV, and V. Ten of the 32 idiopathic HP patients complained of diplopia, indicating cranial nerve III and VI deficits. Cranial nerves II (57%) and VIII (29%) were the most frequently involved nerves in AAV patients. Deficits in cranial nerves II (25%), VII (25%), and VIII (25%) were common in HP patients with tuberculous meningitis. Cranial nerves VI (33.3%) and II (50%) were the only affected nerves in HP patients with viral and bacterial meningitis, respectively.

Other clinical features in the nervous system include seizures, vision loss, diplopia, hearing loss, disturbance of consciousness, sensory disorder, loss of muscle strength, ataxia, and suspicious positive pathologic reflex. Seizures can be found in 2 tuberculous meningitis HP patients (2/4) and 1 idiopathic HP patient (1/32). Vision loss can be found in idiopathic HP (9/32), AAV HP (4/7), and tuberculous meningitis HP (1/4). Diplopia can be found in idiopathic HP (10/32) and viral meningitis HP (1/3). Disturbance of consciousness can only be found in idiopathic HP (2/32). Sensory disorder can be found in one idiopathic HP patient (left facial superficial sensory decreased). Loss of muscle strength can be found in one idiopathic HP patient (right upper and lower limbs, level 3). Ataxia can only be found in idiopathic HP (2/32). Suspicious positive pathologic reflex can be found in idiopathic HP (1/32), AAV HP (1/7), and tuberculous meningitis HP (2/4).

Besides the nervous system, there were also defects in other systems. Fever can be found in all kinds of HP (two in idiopathic HP patients and one in all other HP patients). Pulmonary embolism, deep venous thrombosis, abnormal liver function, or renal function can be found in AAV HP. Obsolete pulmonary tuberculosis can be found in tuberculous meningitis HP. Sinusitis is the most common symptom in idiopathic HP patients, and other symptoms include subclinical hypothyroidism, abnormal liver function, and granuloma of the external auditory canal.

### Laboratory Features

**Figure 3:**
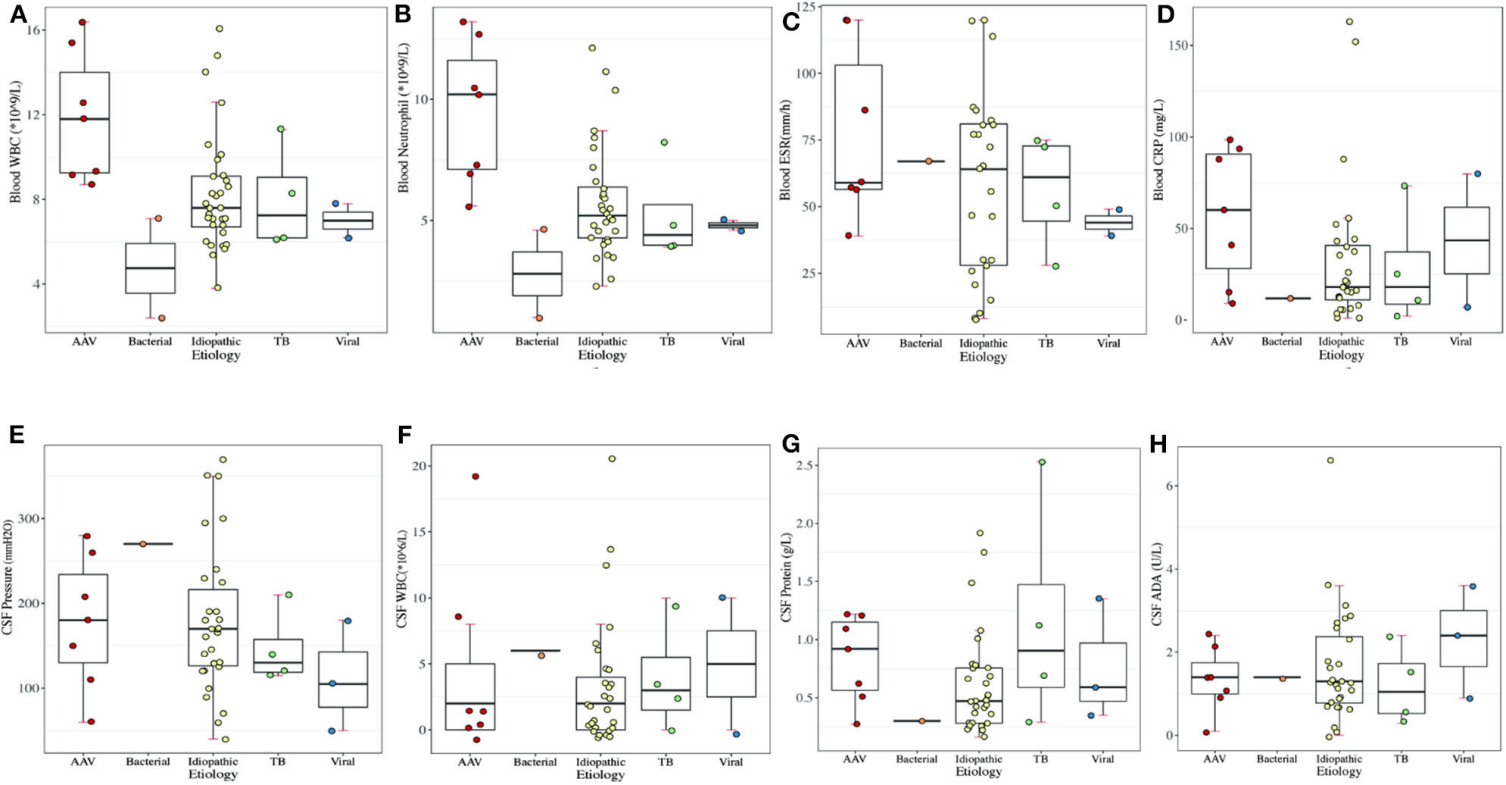
Laboratory results of periphery blood in different etiologies of HP. **(A)** Blood WBC (normal range: 3.5–9.5 × 10^9^/L). **(B)** Blood neutrophil count (normal range: 1.8–6.3 × 10^9^/L). **(C)** Blood ESR (normal range: 0–21 mm/h). **(D)** Blood CRP (normal range: 0–8 mg/L). **(E)** CSF WBC (normal range: 0–8 × 10^6^/L). **(F)** CSF pressure (normal range: 80–180 mm H_2_O). **(G)** CSF protein (normal range: 0.15–0.45 g/L). **(H)** CSF ADA (normal range: <40 U/L).

**Figure 4:**
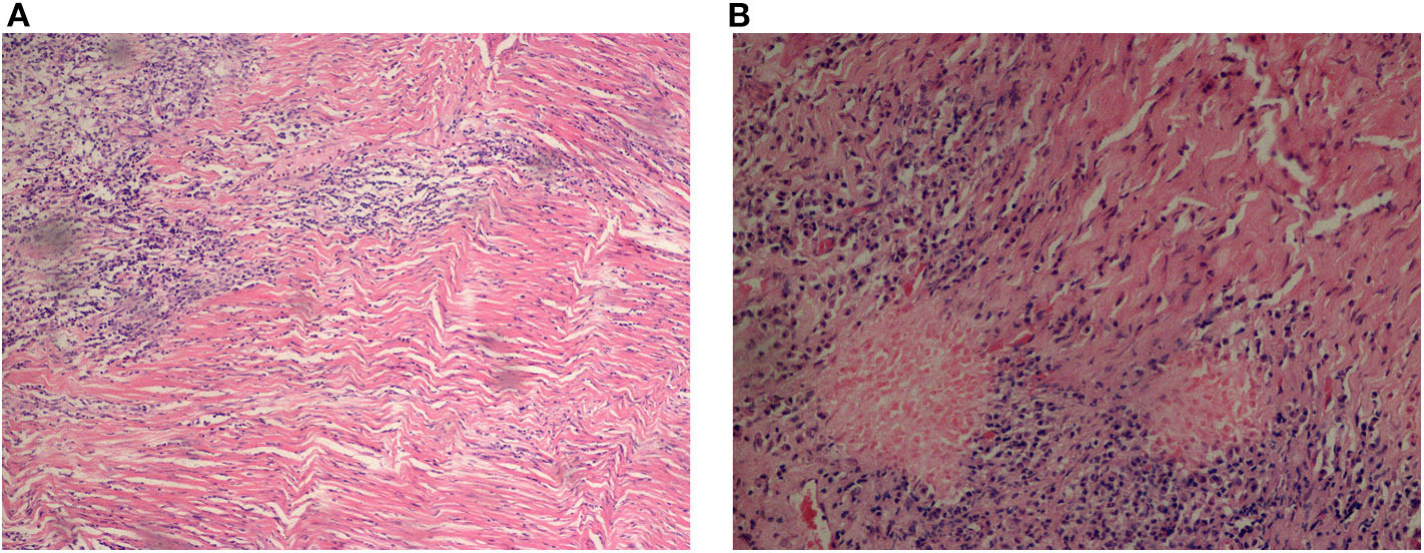
Pathological results of dura mater from an idiopathic HP patient. T cell and B cell infiltration were shown by immunohistochemistry. **(A)** (Right dura mater and underneath) fibrosis and regional hyaline, with chronic inflammatory cell infiltration, 200 ×. **(B)** 400 ×.

Elevated ESR (87.5%) topped the rank of altered laboratory findings in blood tests, followed by CRP elevation (79.0%) (Supplementary Table 1). RF elevation mainly occurred in 4 cases (57.1%) of AAV and 3 cases (9.4%) of idiopathic HP. WBC elevation (57.1%) and neutrophil elevation (85.7%) presented mostly in patients with AAV, accounting for 57.1 and 85.7% of AAV-related HP, respectively. Regarding idiopathic HP and tuberculous meningitis, WBC and neutrophil elevation occurred in ~25% of HP patients. CSF pressure increased in 10 patients with idiopathic HP, followed by 3 patients with AAV and 1 patient with tuberculous meningitis and BM. CSF protein elevation was present in 17 patients with idiopathic HP, 7 patients with AAV, 3 patients with tuberculous meningitis, and 2 patients with viral meningitis (Figure 3). We further looked at the ANCA-related findings (since not all patients were tested, the result was not very completed): antinuclear antibodies (ANA) elevation (2/5), myeloperoxidase (MPO)-ANCA positivity (4/5), proteinase 3 (PR3)-ANCA positivity (4/6), and IgG4 elevation (2/2). Dural biopsy of a patient with idiopathic HP showed fibroplasia and chronic inflammatory cell (mainly T cells and B cells) infiltration (Figure 4).

### Treatment Modalities

Glucocorticoids were the most used drugs and were administered to 68.8% of all patients (33/48). Immunosuppressants were applied to 14.6% of all patients (7/48), only in AAV HP (4/7) and idiopathic HP (3/32). For idiopathic HP patients, 16 patients were receiving active follow-up. Eight of these patients improved, and three relapsed with glucocorticoid treatment, whereas four of them improved, and one relapsed without the use of glucocorticoids. For ANCA-related HP patients, four patients had partial responses, whereas one patient relapsed after treatment with steroids. Two HP patients with tuberculous meningitis progressed despite glucocorticoid treatment. Additionally, two HP patients with viral meningitis and BM recovered after the etiological treatments.

## Discussion

HP is a rare disease worldwide with relatively low prevalence, but that is seriously debilitating or life-threatening. The existing studies of HP are usually based on small sample sizes, especially in China. To our knowledge, this is the largest HP study conducted in China with the aim of exploring the clinical features of people with HP and could be useful for clinicians in diagnosis and prognosis.

We found that the most common type of HP was idiopathic HP, followed by AAV-related HP, tuberculous-associated HP, viral-related HP, and bacterial-related HP. No sex preference was identified in our study, which was not consistent with previous works (2–4). The onset of the first symptoms mainly occurred at an age of 50 ± 12 years, which was earlier than the patients in the US and Japan (2, 5). The definite time to diagnosis of HP ranged from 0.25 to 144 months (with an average of 22 months), among which the HP patients with BM had the shortest duration (0.6 ± 0.4 months), and idiopathic HP patients had the longest (25 ± 36 months). For idiopathic HP patients, many different types of examinations were needed to rule out multiple possible reasons before the final diagnosis, which may explain why idiopathic HP patients required a relatively long time for diagnosis.

In our results, idiopathic, AAV, and viral HP showed “diffuse” enhancement patterns, which was also confirmed by Hahn et al. in idiopathic HP patients. We found that all cases of tuberculous meningitis appeared with “focal” enhancement patterns, which was similar to the results reported from Portugal (7). The “roughness” of the enhancement patterns seems to be a distinguishing factor for idiopathic HP. An “irregular” pattern was common in idiopathic HP patients, whereas other HP patients were likely to present a “regular” pattern. However, Hahn et al. (6) hold a contrasting view that the majority of idiopathic HP patients in the US exhibited regular enhancement instead of an irregular pattern. The reasons for the different conclusions between the two studies are not clear. The question of whether race was taken into consideration will require more studies in the future.

Locations of the thickened dura mater differ among different causes. The posterior fossa was the most affected area in idiopathic HP and tuberculous meningitis HP according to our study, which was also reported in other studies (6, 8, 9). It is notable that posterior fossa HP can cause acute non-communicating hydrocephalus (9). In addition to the posterior fossa, thickened falx cerebri (10–14), anterior cranial fossa (15), cerebellar tentorium (10, 16), frontal lobes (10, 11, 17), sphenoid wings (18), cavernous sinuses (19), and paranasal maxillary sinuses (19, 20) were also involved in HP patients. With enhancements in the cerebral falx and tentorium cerebelli in coronal scanning, a typical “Eiffel Tower” (21, 22) or “Benz” sign emerged (23). Enhancement in the cerebral falx or tentorium cerebelli can be mistakenly regarded as subarachnoid hemorrhage or superior sagittal sinus. Thus, attention must be paid to the signs in images to avoid misdiagnosis. Temporal or occipital enhancements may be secondary to suppurative otitis media. Granulation or effusion in the middle ear caused by small vasculitis may spread to the dural mater and lead to secondary HP (4). Therefore, patients with otitis media or concurrent multiple cranial nerve deficits should receive contrast-enhanced MRI to rule out HP. In addition, the involvement of the junction of the craniocervical area has been reported in several cases (8, 24) and causes obstructive hydrocephalus along with cerebellar tonsillar herniation. Peripheral to the lesion, there is usually a circle of enhancement in images (18, 25), which may be caused by a zone of active inflammation along with the border of the lesion (5). Enhancement of spinal HP is typically found within the cervical and high thoracic regions. In our study, the lumbar and cervical dura was enhanced in several idiopathic HP patients. In a Japanese retrospective study, dura mater in the convexity was the most commonly thickened area in AAV-induced HP (26). These findings suggested the potential role of racial differences for differential diagnosis in dural enhancement patterns in HP.

Headache was the most common clinical feature of HP, regardless of etiology (6), and was also the case in our study. As an initial symptom of HP, headache can progress over time (12). Thus, for patients with progressive chronic headache, enhanced MRI is of great importance. Locations of headache mainly coincided with the locations of enhanced dura mater in MRI (27), which suggested that headache might be associated with irritations caused by inflammation (18), stenosis of cerebral venous sinus resulting from fibrosis (28), and hydrocephalus and intracranial hypertension caused by difficulty reabsorbing CSF (29). Cranial nerve deficits, the second most common symptom of HP, might be caused by oppressive ischemia, thickened meninges encroachment, or epineurium inflammatory infiltration (30). Cranial nerve deficits can be divided into two types according to the location of the thickened dura mater. Cranial nerve II to VI deficit might be associated with thickened meninges from the cavernous sinus to the superior orbital fissure. Thickened meninges from the cerebral falx and tentorium cerebelli to the posterior cranial fossa may lead to cranial nerve IX to XII dysfunction. Cranial nerves II and VIII were the most affected cranial nerves in HP, which was in agreement with our study (6). Other common symptoms in HP patients in our study included psychiatric disorders (12, 18), ataxia, and seizures (31), which were in accordance with previous studies. Venous sinus thrombosis (32–34) and venous sinus stenosis (35–37) were reported in some Western countries but have not been identified in China, including in our study (38). Idiopathic hypertrophic spinal pachymeningitis (IHSP) mainly affects the cervical and thoracic spine (86%) and, in rare situations, involves the entire spine (7%) (8). Only a few cases of the craniospinal form of idiopathic HP have been reported (24, 39). Generally, the ventral spine canal dura was more susceptible than the dorsal spine canal dura (18). The most common symptom, paralysis, occurred in 71% of the HSP patients. Other symptoms include numbness (64%), bladder and rectal dysfunction (43%), back pain, and nerve root or spinal cord compression (8).

In a retrospective study of 12 Chinese patients, elevated CRP and ESR were reported (38), which is in accordance with our results, revealing an inflammatory nature of HP. In a retrospective study of 22 Chinese patients diagnosed with HP, 17 patients had abnormal CSF and/or abnormal biochemical tests, such as increased CSF pressure and elevated CSF protein levels and immunoglobulins (10), which was also in line with our study. Blood–CSF barrier damage caused by adjacent inflammation, such as necrotizing vasculitis at the arachnoid, enabled inflammatory cell infiltration and increased immunoglobulin levels in the CSF (40). Excessive proteins may be synthesized by intrathecal plasma cells due to a fibroinflammatory immune reaction (2) to stabilize the CSF internal environment. The arachnoid is involved in idiopathic HP, and a proportion of immunoglobulins may originate from the blood because of damage to the blood–CSF barrier at the arachnoid (30).

According to a large-scale national epidemiological survey of 159 cases that was conducted in Japan, the majority of HP cases were idiopathic (44%). The prevalence rates of ANCA-related HP and IgG4/multifocal fibrosclerosis (MFS)-related HP were 34.0 and 8.8%, respectively (2). In our study, the highest proportion was idiopathic HP, followed by AAV-related HP and infection-related HP. No cases of IgG4-related HP were confirmed in our study since few of them underwent biopsies. Immune-related HP included HP caused by AAV, IgG4-related disorder, rheumatoid arthritis, sarcoidosis, Behcet disease, and Sjogren syndrome. In a retrospective study in Japan, 7 of the 39 patients with AAV presented with HP (41). AAV includes granulomatosis with polyangiitis (GPA), microscopic polyangiitis (MPA), and eosinophilic granulomatosis with polyangiitis (EGPA). GPA, characterized by necrotizing granulomatous vasculitis in small-to-medium vessels, mainly affects the respiratory tract, pulmonary capillaries, and kidneys (42). Additionally, central nervous system involvement has been reported in 22–54% of GPA cases (43, 44). MPA is a systemic vasculitis of small-to-medium-sized vessels that is associated with antibodies directed against the target antigen MPO (45). GPA is the most common form of AAV-related HP, followed by MPA. In a recently published article, the HP incidence was significantly higher in patients with GPA than in those with MPA (60.2 vs. 3.3 persons per 1,000 person-years, respectively) (46). The reasons why AAV-related disorders affect the CNS may include the following: (1) granulomatosis tissue extends directly to the intracranial nervous system from adjacent lesions in the orbit or the paranasal cavity, (2) granulomatosis tissue transfers to the intracranial nervous system from the respiratory tract, and (3) vasculitis affects intracranial vessels (29).

In addition, IgG4-related disorder (IgG4-RD) is a newly recognized immune-mediated fibroinflammatory disease that affects multiple systems. Th1–Th2 balance is an interesting topic in IgG4-RD. Early on, it was discovered that ANCA-related HP shows Th1 shift in the CSF (1). Later, a Th2 cytokine shift was also identified in the CSF of IHP patients, which is relevant to plasma cell infiltration in idiopathic and IgG4-related HP (47). T follicular helper (Tfh) cells are a subset of CD4+T cells that are known to be involved in the differentiation and class switch of B cells during their development. Among Tfh cells subsets, Tfh2 cells could induce the differentiation of naïve B cells into plasmablasts, subsequently promoting the production of IgG4 in active (48). Glucocorticoid-induced remission in patients with IgG4-RD is associated with a decrease of circulating CD4+ CTLs, a recently identified potentially pathogenic population of T lymphocytes (49). PET/CT represents a reliable instrument for assessing IgG4-RD activity (50). The histopathological features of IgG4-related HP include lymphoplasmacytic infiltration of IgG4-positive plasma cells, storiform fibrosis, and obliterative phlebitis. Diagnosis may be challenging, given that serum IgG4 concentrations were neither specific nor sensitive and biopsy samples stained for IgG4+ plasma cells were difficult to obtain. Our AAV HP patients did not have a meningitis biopsy, and only two of seven patients have CSF IgG results. According to a study, CSF IgG4 quantification and IgG4 Indices serve as alternatives to meningeal biopsy for the diagnosis of IgG4-HP when this procedure is contraindicated or uninformative (51). Treatment for IgG4-related HP is under controversy. Systemic administration might not be as effective on putative pathogenic B cells residing in inflammatory niches within the CNS. Intrathecal rituximab was reported to be effective (52).

Infection-related HP usually originates from peri-cranial infections, such as paranasal sinusitis, otitis media (29), or mastoid process inflammation (12). Common pathogens include *Mycobacterium tuberculosis, Treponema pallidum*, and Epstein–Barr (EB) virus (18). Uncommon infections, including Lyme disease, cysticercosis, and human T cell lymphotropic virus, are also involved in the pathogenesis of HP (5). However, it has also been reported that untreated HP is a predisposing factor for BM due to *Streptococcus pneumoniae* (53). More efforts should be made to draw a cause–effect conclusion.

The histopathology of HP shows infiltration of small mature lymphocytes, plasma cells, and epithelioid histiocytes at the surface of the dura mater (25). Dense fibrosis occurs, mainly consisting of collagen fibers associated with hyaline degeneration, arranged in a concentric-circle-like manner (31). This can be explained by a theory that inflammatory infiltrate activates fibroblasts and induces collagen deposition, leading to tissue hypertrophy and increased dural thickness (54). Necrotizing vasculitis of small arteries located in the dura and cerebral surfaces has also been reported (12). In our study, only one patient underwent a biopsy, which was mainly conducted to confirm the diagnosis of IgG4-RD (54–56). In fact, the biopsy rates were low in both China and other countries because of the potential risks of the procedure. Empiric treatment was usually administered when there was sufficient clinical evidence without biopsy results (4, 23).

Etiological treatments, including antibiotic, antifungal, and antituberculosis drugs, are essential in HP patients. Glucocorticoids are considered the first-line therapy after the exclusion of infection. However, consensus on the course and dose of glucocorticoid treatment has not been reached. The classical treatment for HP patients in the active stage is methylprednisolone pulse therapy (500 mg/d for 3 days), followed by maintenance treatment with oral prednisone. The maintenance is also important since disease recurrence occurred when prednisone was reduced to 10–30 mg/d (57); the condition can also sometimes cause progressive deterioration and death (18).

Disease recurrence is one of the major concerns in treatment. Approximately 50% of HP patients are reported to have disease relapse (4). In our study, three idiopathic HP patients relapsed on a 30–50 mg of prednisone daily dose, and an ANCA-related HP patient suffered a relapse at a dose of 30 mg of prednisone, suggesting that the corticosteroid should be tapered off extremely slowly in case of recurrence. For refractory HP patients, long-term steroid monotherapy may lead to potential adverse effects as well as disease recurrences. Immunosuppression therapies, including azathioprine (58, 59), cyclophosphamide (58, 60), rituximab, or combined therapies, are often therapeutic options or adjuvant treatments for steroids. Rituximab (RTX), a monoclonal antibody targeting CD20 on the surface of pan-B cells, selectively suppresses B-cell-associated autoimmunity. RTX treatments for HP with IgG4-RD suppress the reciprocal activation of T2-helper cells to relieve the systemic inflammation (57). Three steroid-refractory HP patients treated with RTX for 4 weeks showed clinical improvements and exhibited prominent decreases in dural thickness (61). Thus, RTX has been suggested to be a second-line therapy for steroid-refractory HP, especially for IgG4-RD (52–65).

Several limitations associated with the present study warrant mention. First, the single-center study does not allow generalization for southern China. Second, the retrospective nature of the study means that it might lack reliability. Third, due to the low rate of histology, the idiopathic HP patients might be elevated, since some of the idiopathic HP patients might be categorized into other etiology with pathology results.

In conclusion, HP is a rare fibrosing inflammatory disorder characterized by localized or diffuse thickening of the cranial or spinal dura mater. In our study, the etiology of HP included a wide spectrum of conditions. Idiopathic HP accounted for the majority, and AAV-related HP was the most frequent form of secondary HP in the southern Chinese population. The most common symptom was headache, followed by cranial nerve deficits. Elevated ESR was the most frequently changed laboratory finding in HP patients, followed by CRP. MRI with contrast showed that the thickening of the dura mater differs according to different etiologies. Glucocorticoid or immunosuppressive agents were the primary drugs used in the treatment of HP patients.

## Data Availability Statement

The original contributions presented in the study are included in the article/Supplementary Material, further inquiries can be directed to the corresponding author.

## Ethics Statement

The studies involving human participants were reviewed and approved by IRB of Xiangya Hospital. The patients/participants provided their written informed consent to participate in this study.

## Author Contributions

XX and LF conceived the presented idea. DF and XX collected the data and wrote the manuscript. DF performed the computations. All authors discussed the results. All authors listed have made a substantial, direct and intellectual contribution to the work, and approved it for publication.

## Conflict of Interest

The authors declare that the research was conducted in the absence of any commercial or financial relationships that could be construed as a potential conflict of interest.
