# Supplementary material for: Hypertrophic Pachymeningitis in a Southern Chinese Population: A Retrospective Study
Source: Front Neurol. 2020 Nov 17;11:565088. doi: 10.3389/fneur.2020.565088 (PMC7705170; doi:10.3389/fneur.2020.565088)
Supplement: Supplementary file 1 [file Table_1.DOCX]

**Supplementary Table 1 Patient clinical features, laboratory results, treatment, and follow-up.**

| Diagnosis | N | Age at presentation±STDEV | Gender | Duration (month) | Clinical presentation | ESR elevation | CRP elevation | CSF pressure elevation | CSF protein elevation | CSF glucose decrease | CSF ADA elevation | CSF immune globulin elevation | Biopsy features | Treatment modalities | Follow-up |
| --- | --- | --- | --- | --- | --- | --- | --- | --- | --- | --- | --- | --- | --- | --- | --- |
| Idiopathic HP | 32 | 49.4±11.9 | 20M 12F | 25±36 | Headache（30）  Cranial nerve deficits (17)  Disturbance of consciousness(2) Fever(2)  Ataxia (2) Seizure (1) | 28/32 | 26/32 | 10/30 | 17/30 | 5/30 | 0 | IgA(8/23) IgG(19/23) IgM(6/23) | fibroplasia and chronic inflammatory cell infiltration (1) | Steroids (22) Mycophenolate Mofetil (1) Azathioprine (2) | 16 patients with active follow-up;8 patients improved and 3 patients relapsed after the treatment; 4 patients improved and 1 patient relapsed without the use of steroid and other immunosuppressors |
| ANCA-associated vasculitis | 7 | 59.4±9.1 | 2M 5F | 14±16 | Headache（7）  Cranial nerve deficits (4) Visual acuity decreased （3） Fever(1) Hearing loss (1)  Dizziness (1) Diuresis (1) Arteria temporalis eminence (1) Cushing sign (1) Pathologic reflex suspicious positive (1) | 7/7 | 7/7 | 3/7 | 7/7 | 1/5 | 0 | IgA(3/4) IgG(4/4) IgM(2/4) | - | Steroids (5) Mycophenolate Mofetil (2) Cyclophosphoramide (4) | 5 patients with active follow-up; 4 patients with partial response while 1 patient relapsed after the treatment |
| Tuberculous meningitis | 4 | 51.0±5.6 | 0M 4F | 33±50 | Headache（3） Cranial nerve deficits (3) Pathologic reflex positive (2) Neck pain (1) Visual acuity decreased （1）Fever(1) Seizure (1) Vomiting （1） Meningeal irritation sign （1） |  | 3/4 | 1/4 | 3/4 | 0/4 | 0 | IgA(3/3) IgG(3/3) IgM(2/3) | - | Steroids（2） Anti- Tuberculous therapy (1) | 2 patients progressed and 2 patients were lost to follow-up after the treatment |
| Viral meningitis | 3 | 37.7±4.6 | 0M 3F | 0.7±0.2 | Headache（3） Cranial nerve deficits (1) Fever(1) Vomiting （1） Palpitation （1） Photophoby（1） Meningeal irritation sign （1） | 2/2 | 1/2 | 0/3 | 2/3 | 2/3 | 0 | - | - | Anti-virus therapy (3) | 1 patient completely recovered and 2 patients were lost to follow-up after the treatment |
| Bacterial meningitis | 2 | 46.5±19.5 | 2M 0F | 0.6±0.4 | Headache（1） Syncope (1) Cranial nerve deficits (1) Disturbance of consciousness(1) Visual acuity decreased （1） Cognitive disorder (1) Hearing loss (1) | 1/2 | 1/2 | 1/2 | 0/2 | 1/2 | 0/2 | - | - | Steroids (1) | 1 recovered completely and 1 patient was lost to follow-up after the use of steroid |

Abbreviations: N:number STDEV: standard deviation
